# Supplementary material for: Complexome profiling of the Chlamydomonas psb28 mutant reveals TEF5 as an early PSII assembly factor
Source: Plant Cell. 2025 Mar 17;37(6):koaf055. doi: 10.1093/plcell/koaf055 (PMC12164590; doi:10.1093/plcell/koaf055)
Supplement: koaf055_Supplementary_Data [file koaf055_supplementary_data.zip › psb28_interactive.zip!/plots/intensity/qualityPlots/peptides/_Cre12.g534250.t1.2.html]

### Cre12.g534250.t1.2

Trivial name:   
SeqCoverage [median]: 46.90  
Mass: 23.75 kDa  
  
 

| Sequence | MissedCleavage | Mass [Da] | wtIntensity | muIntensity | Score |
| --- | --- | --- | --- | --- | --- |
| ALSVFEATGK | 0 | 1021.54 | 7827.09 | 5948.00 | 92.44 |
| ALSVFEATGKTSKEETLR | 2 | 1966.04 | 0.00 | 929.90 | 80.64 |
| EDFVLDAQAK | 0 | 1134.56 | 0.00 | 508.53 | 87.31 |
| FRPFQYIPVK | 1 | 1293.72 | 0.00 | 388.90 | 34.72 |
| KALSVFEATGK | 1 | 1149.64 | 1901.56 | 3365.64 | 82.44 |
| LVDQETVEGAILHMER | 0 | 1838.92 | 13518.83 | 45020.22 | 158.64 |
| LVFGTATK | 0 | 835.48 | 3224.95 | 12119.65 | 89.75 |
| SGPTPDIAGR | 0 | 969.49 | 13346.77 | 9008.04 | 113.71 |
| SSAGGVALLIK | 0 | 1014.61 | 25350.03 | 55493.23 | 247.75 |
| VDIHVLGEQK | 0 | 1136.62 | 0.00 | 9478.13 | 130.94 |
|  |  |  | 65169.2 | 142260.2 |  |
